# Supplementary material for: Differential Marker Expression between Keratinocyte Stem Cells and Their Progeny Generated from a Single Colony
Source: Int J Mol Sci. 2021 Oct 6;22(19):10810. doi: 10.3390/ijms221910810 (PMC8509450; doi:10.3390/ijms221910810)
Supplement: Supplementary file 1 [file ijms-22-10810-s001.zip › Supplementary figure S1.pptx]

## Slide 1
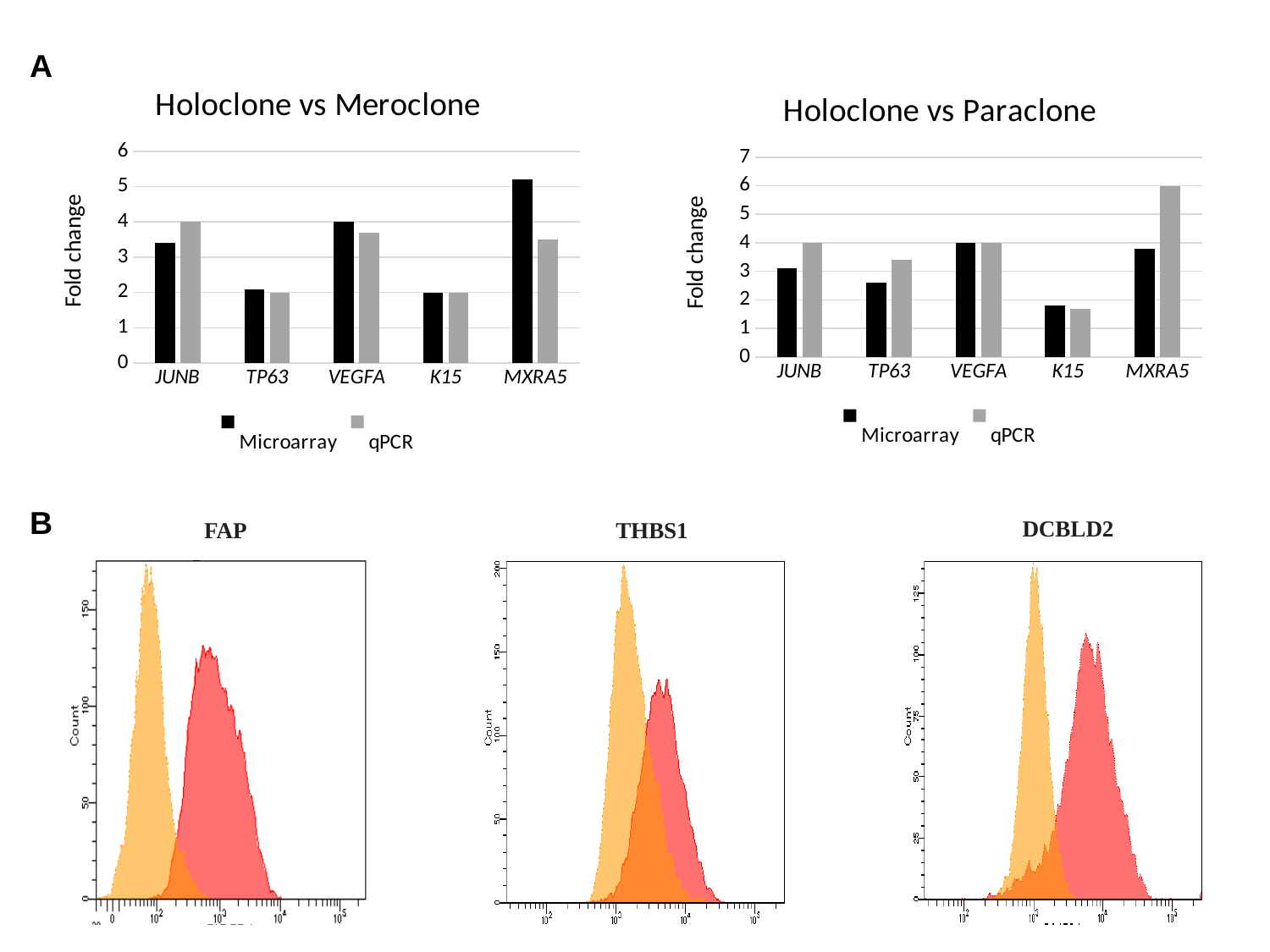

A
### Chart: Holoclone vs Meroclone
| Category |
Microarray |
qPCR |
|---|---|---|
| JUNB | 3.4 | 4.0 |
| TP63 | 2.1 | 2.0 |
| VEGFA | 4.0 | 3.7 |
| K15 | 2.0 | 2.0 |
| MXRA5 | 5.2 | 3.5 |
### Chart: Holoclone vs Paraclone
| Category |
Microarray |
qPCR |
|---|---|---|
| JUNB | 3.1 | 4.0 |
| TP63 | 2.6 | 3.4 |
| VEGFA | 4.0 | 4.0 |
| K15 | 1.8 | 1.7 |
| MXRA5 | 3.8 | 6.0 |B
DCBLD2
FAP
THBS1
