# Supplementary material for: Differential Marker Expression between Keratinocyte Stem Cells and Their Progeny Generated from a Single Colony
Source: Int J Mol Sci. 2021 Oct 6;22(19):10810. doi: 10.3390/ijms221910810 (PMC8509450; doi:10.3390/ijms221910810)
Supplement: Supplementary file 1 [file ijms-22-10810-s001.zip › Supplementary table S4.pdf]

Supplementary Table S4. Significantly enriched canonical pathways.

Top canonical pathways based on significance	-log (P-value)	z-score
**Holoclone vs Meroclone**		
Aldosterone signaling in epithelial cells	6.5	Activated
P53 Signaling	6.3	Inhibited
Cell cycle: G1/S cell cycle checkpoint regulation	4	Activated
VDR/RXR activation	3.8	Inhibited
Super pathway of serine and glycerin biosynthesis I	3	Activated
EPK/MAPK signaling	3	Activated
Ketogenesis	2.5	Activated
Oxidative ethanol degradation III	2.5	Activated
VEGF signaling	1.5	Inhibited
**Holoclone vs Paraclone**		
EIF2 signaling	9.5	Activated
NER pathway	9.2	Activated
P53 signaling	7.5	Inhibited
mTOR signaling	7.2	Activated
Super pathway of serine and glycerin biosynthesis I	3	Activated
Oxidative ethanol degradation III	3	Activated
HGF signaling	2.5	Activated
Histamin degradation	2.5	Activated
Melanoma signaling	2	Activated
EPK/MAPK signaling	2	Activated
ErbB4 signaling	2	Activated
ERK5 signaling	1.5	Activated
VEGF signaling	1.5	Activated
Ketogenesis	1.5	Activated
NGF signaling	1.5	Activated
**Meroclone vs Paraclone**		
Role of BRCA1 in DNA damage response	12	Activated
NER Pathway	10	Activated
Mitotic rule in polo like kinase	7.5	Activated
Purine nucleotides de novo biosynthesis II	7.5	Activated
Estrogen mediate S phase entry	5	Activated
Cell cycle: G2/M DNA damage cell cycle checkpoint regulation	5	Inhibited
Cell cycle: G1/S cell cycle checkpoint regulation	5	Inhibited
